# On-Orbit Modulation Transfer Function Estimation Based on the Refined Image Kernel

**DOI:** 10.3390/s23094362

**Published:** 2023-04-28

**Authors:** Yuanhang Wang, Xing Zhong, Zheng Qu, Lei Li, Sipeng Wu, Chaoli Zeng

**Affiliations:** 1 Changchun Institute of Optics, Fine Mechanics and Physics, Chinese Academy of Sciences, Changchun 130033, China; wangyuanhang19@mails.ucas.ac.cn (Y.W.);; 2 University of Chinese Academy of Sciences, Beijing 100049, China; 3 Chang Guang Satellite Technology Co., Ltd., Changchun 130102, China; 4 Key Laboratory of Advanced Technology for Aerospace Vehicles of Liaoning Province, Dalian University of Technology, Dalian 116024, China; 5 State Key Laboratory of Structural Analysis for Industrial Equipment, Dalian University of Technology, Dalian 116024, China

**Keywords:** remote sensing, MTF, refined image kernel, energy concentration, image quality assessment

## Abstract

To overcome the limitations of traditional on-orbit modulation function transfer (MTF) measurement methods that are heavily dependent on natural features, scenery, artificial edges, and point source targets, this paper presents an on-orbit MTF measurement method of remote sensing imager based on the refined image kernel (RIK) acquired directly from remote sensing images. First, the kernel is estimated from some remote sensing sub-images with rich texture details by using an iterative support detection (ISD) algorithm; then, it is refined by central pixel energy concentration (EC) to obtain the RIK. Secondly, the MTF curves are calculated by interpolating RIK and Fourier transform. Finally, the final MTF is the average value of MTFs at Nyquist frequency acquired by each RIK. To demonstrate the feasibility and validity of this method, the MTFs were compared to the result of the ISO12233 edge method with an error of no more than 7%. The relative error of the measured results does not exceed 5% for image signal-to-noise ratio (SNR) above 20dB. The results obtained from the on-orbit MTF measurement using remote sensing images of the Jilin-1 satellite have a maximum error of less than 2% compared with the ISO12233 edge method. These demonstrate that the method proposed in this paper supplies highly accurate and robust results and can successfully increase the efficiency of on-orbit MTF measurement, providing a reference for high-frequency monitoring of satellite on-orbit stability and their optical imaging quality.

## 1. Introduction

High-resolution optical remote sensing satellites have undergone extensive development in recent years as a result of the continuous advancements in science and technology, and the resulting high-resolution remote sensing images have extremely broad application potential as well as significant values in fields such as natural resource analysis, ecological environmental protection, and geographic mapping [1,2,3,4]. When discussing the imaging performance of an optical remote sensing camera, a crucial component of a remote sensing satellite, the MTF is typically employed to characterize the response of an imaging system to various spatial frequency input signals [5,6,7]. Although the camera is calibrated and evaluated in the lab before launch, the actual on-orbit MTF fluctuates to variable degrees due to factors including launch-related vibrations and the space environment [8,9,10,11]. Therefore, it is necessary to measure the on-orbit MTF to monitor the actual performance of on-orbit cameras.

The on-orbit MTF of the remote sensing imager is typically obtained by digital Fourier analysis, which is based on the object–image correlation of the optical system. The captured image and the chosen reference target characteristics are then digitally processed to extract the on-orbit MTF, which is currently available in a variety of methods, including the knife-edge method, the point light source method, the pulse method, and the periodic target method [12,13,14,15,16,17]. These methods are used by high-resolution satellites, including QuickBird, IKONOS, SPOT, Landsat, and GF, for on-orbit MTF measurements. Each technique has its benefits and has shown itself to be somewhat successful [18,19,20,21,22]. However, it is challenging to detect ideal knife edges and point sources in natural features, and because knife edges lack a frequency component, additional line spread function (LSF) extraction is required to resolve the frequency information, and this process is easily impacted by the contrast and noise of natural characteristics, which increases errors and reduces the accuracy of on-orbit image quality assessment [23]. Identifying optimal pulsed targets is difficult due to divides in satellite resolution, and it can take some time to locate pulsed natural features in a fixed direction. Additionally, the pulse width can have an impact on measurement accuracy. Point source targets require strict control of the irradiance of the light source to prevent overexposure and affect the accuracy of MTF measurement. Manual targets require labor-intensive setup, and the amount of data that can be caught by MTF detection in the fixed direction of a periodic target is insufficient. The time required to acquire MTFs and the expense of on-orbit MTF measurements grow due to the limits of specific orbits and revisit periods of remote sensing satellites.

In this paper, first, an approach of extracting image kernels is employed to establish and illustrate how to estimate the MTF by image kernels; the fundamentals of the kernel estimation algorithm and the calculation of the MTF are presented in Section 2. Secondly, in Section 3, the factors affecting the accuracy of kernel estimation are given, and the refinement of the image kernel by energy concentration to obtain a refined image kernel is investigated. Then, the validation experiments for the suggested method are thoroughly described, and the effects of dynamic MTF levels and image SNR on measurement outcomes are also covered. These contents are detailed in Section 4 and Section 5. Finally, the method proposed in this paper is applied to the measurement of the on-orbit MTF of the Jilin-1 satellite remote sensing imager, and the comparison of the measurement results with those obtained by the conventional method is also analyzed in Section 6.

The highlights of this paper are as follows: (1) A method for measuring the on-orbit MTF of remote sensing satellite optical payloads is proposed, which does not rely on feature scenery (such as rooflines, farmlands, roadways) or artificially established edges and point source targets, and extracts the image kernel directly from remote sensing images with rich texture details, and estimates the MTF by the refined image kernel obtained from refining the kernel using energy concentration; (2) the method greatly simplifies the process of measuring the on-orbit MTF. In theory, only a cloud-free detail-rich remote sensing image is needed to estimate the on-orbit MTF of a remote sensing satellite imager, and the measurement result has only a small error compared with the traditional method; (3) this method can achieve high-frequency measurement and real-time monitoring of the on-orbit imaging quality of optical remote sensing satellites.

## 2. Fundamentals of Measurement

### 2.1. Modeling of Modulation Transfer Function

**Figure 1:**
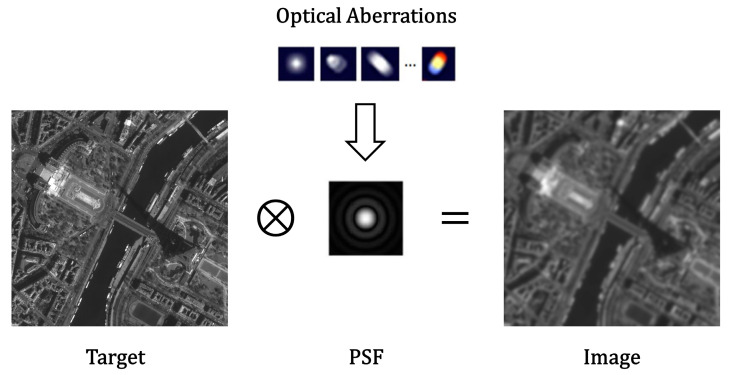
Model of optical remote sensing camera imaging procedure.

Optical remote sensing imagers can be thought of as linear systems. It is feasible to model the imaging procedure as a convolution operation. The relation between the background object and the image is usually described (illustrated in Figure 1) as

(1) g(x,y)=o(x,y)⊗k(x,y)+ε

where (x,y) is the coordinate on a continuous spatial domain, g(x,y) denotes the image captured by the camera, o(x,y) is the objective scene, k(x,y) is the kernel, which is an imaging system can be thought of as the point spread function (PSF) of the optical system, ε indicates the image noise, and ⊗ represents the convolution operation.

Point spread function can be used to describe the blurring of an optical remote sensing camera when acquiring an image, etc., for an imaging system where the input is an ideal point source; the output is no longer a point but a diffused spot. The kernel extracted from the image is interpolated and fitted to obtain the system PSF, and then the Fourier transform is carried out to obtain the optical transfer function (OTF) is

(2) OTF(u,v)=∬k(x,y)exp[−2iπ(xu+yv)]dxdy

where u and v are the spatial frequencies in the frequency domain along the two coordinate directions, respectively. The MTF can be expressed as

(3) MTF(u,v)=|OTF(u,v)|

since the MTF calculation using a single kernel may have a relatively large error (for reasons mentioned in Section 3), we extract the kernel from serval sub-images and calculate the MTF separately and then average them to obtain the final measured MTF as

(4) $$MTF=\frac{\sum_{i=1}^{n}MTF_{i}}{n}$$

### 2.2. Kernel Estimation Method

Estimating the kernel from the image is crucial for the on-orbit MTF measurement method provided in this study. The image kernel is mostly adopted in blind image deblurring, i.e., estimating the blur kernel and the potential clear image from the input blur image. However, this is an ill-posed problem as there can be infinite pairs of blur kernels and latent images to generate the same blurred image. Therefore, sparse prior and regularization have been given as options for the ill-posed optimization problem, such as Maximum A Posteriori (MAP), $L_{0}$ regularized prior, dark channel prior, learned image prior using a CNN, and Gaussian prior [24,25].

In this regard, we evaluate the kernel using the iterative support detection (ISD) algorithm [26], which consists of two steps. Without imposing much sparsity, the first step seeks to efficiently compute a coarse form of the kernel. Although non-convex optimization is used in the second phase, the initial kernel estimation from step one is carried over.

#### 2.2.1. Estimation of the Initial Kernel

To direct the initial kernel, we filter the image and predict the salient edges. To obtain meaningful step edges, we first pre-smooth the image using Gaussian filtering and then solve the following shock filtering partial differential equation problem as

(5) $$\partial I/\partial t=-sign(\Delta I)\left\|\nabla I\right\|\;,\;I_{0}=G_{0}⊗I_{input}$$

where $\nabla I=(I_{x},I_{y}{)}^{\prime }$, $\Delta I=I_{x}^{2}I_{xx}+2I_{x}I_{y}+I_{y}^{2}I_{yy}$ denote the first- and second-order spatial derivatives correspondingly, and $I_{0}$ indicates the Gaussian smoothed input image, which is used as the starting input for iteratively updating ∂I/∂t.

According to previous research [27], salient edges may not always contribute to the estimation of the kernel, and if the kernel scale is greater than the object scale, the edge information of the image may be detrimental to the kernel estimate process. To properly assess the kernel, the edge information in the image must be filtered, and the availability of edges in the image can be determined by the edge confidence level as

(6) $$R(x)=\frac{\left\|\sum_{y\in N_{h}(x)}\nabla B(y)\right\|}{\sum_{y\in N_{h}(x)}\left\|\nabla B(y)\right\|+0.5}$$

where B is the input image and $N_{h}(x)$ is a window of size h×h centered on the pixel x. The 0.5 is set to avoid an illogical image flattening region. The signed ∇B(y) will mainly cancel out in $\left\|\sum_{y\in N_{h}(x)}\nabla B(y)\right\|$ for thin elements (peaks). The level of image structure information in the $N_{h}(x)$ is estimated by $\sum_{y\in N_{h}(x)}\left\|\nabla B(y)\right\|$, which stands for the sum of the absolute gradient magnitudes of image B in the window. A tiny R suggests the presence of either spikes or a flat region, which inactivates some gradient components. Then, a mask was used to exclude pixels within small R(x) windows as

(7) $$M=H(R-\tau_{r}),$$

where H( · ) denotes the Heaviside step function, producing zeros for negative values and ones for other values, and $\tau_{r}$ is a defined threshold. For kernel estimation, the ultimate salient edges selected are

(8) $$\nabla I^{s}=\nabla \tilde{I}\cdot H(M{\left\|\nabla \tilde{I}\right\|}_{2}-\tau_{s}),$$

where $\tilde{I}$ is the image that the shock filter has processed and $\tau_{s}$ denotes a threshold of the gradient magnitude. By excluding part of the gradients through Equation (6), the accuracy of the kernel estimation is improved. During the iteration, the values of $\tau_{r}$ and $\tau_{s}$ are initially 0.1 and 0.05 and decrease (divide by 1.1 in each iteration) to obtain more edge information. The initial kernel estimation is achieved using the image edges detection and filtering described above, then the objective function with a Gaussian regularizer is described as

(9) $$E(k)={\left\|\nabla I^{s}⊗k-\nabla B\right\|}^{2}+\gamma {\left\|k\right\|}^{2},$$

where γ is a weight. We perform FFTs on all of the variables, set the derivative k to zero, then obtain

(10) $$k={ℱ}^{-1}\left(\frac{\bar{ℱ(\partial_{x}I^{s})}ℱ(\partial_{x}B)+\bar{ℱ(\partial_{y}I^{s})}ℱ(\partial_{y}B)}{ℱ{\left(\partial_{x}I^{s}\right)}^{2}+ℱ{\left(\partial_{y}I^{s}\right)}^{2}+\gamma }\right),$$

where ℱ( · ) and ${ℱ}^{-1}(\;\cdot \;)$ represent the FFT and inverse FFT, respectively. $\bar{ℱ\left(\;\cdot \;\right)}$ is the complex conjugate operator.

Predicting the latent image I of the following layer in the image pyramid by using the previously calculated significant edge gradient as a spatial prior, the objective function is

(11) $$E(I)={\left\|I⊗k-B\right\|}^{2}+\lambda {\left\|\nabla I-\nabla I^{s}\right\|}^{2},$$

where the regularization parameter $\lambda =2e^{-2}$. A few algebraic procedures in the frequency domain yield the following results:

(12) $$I={ℱ}^{-1}\left(\frac{\bar{ℱ(k)}ℱ(B)+\lambda \bar{ℱ(\partial_{x})}ℱ(I_{x}^{s})+\bar{ℱ(\partial_{y})}ℱ(I_{y}^{s})}{\bar{ℱ(k)}ℱ(k)+\lambda (\bar{ℱ(\partial_{x})}ℱ(\partial_{x})+\bar{ℱ(\partial_{y})}ℱ(\partial_{y}))}\right)$$

#### 2.2.2. Kernel Elaboration Based on the ISD Algorithm

After the initial kernel estimation, the procedure is continued using an iterative support detection (ISD) method to ensure that the elements are maximal in each iteration and to overcome the effect of noise on the kernel estimation process. By easing the regularization penalty so that these elements are not considerably impacted by the regularization at each refinement, the approach decreases inaccurate estimates and converges quickly [28].

Each iteration of the ISD begins with the previously estimated kernel $k^{i}$ as a partial support, with the vast elements placed in $S^{i+1}$ and the others relating to $\bar{S^{i+1}}$. $S^{i+1}$ is generated by

(13) $$S^{i+1}\leftarrow \left\{j:k_{j}^{i}>\varphi^{s}\right\}$$

where j stands for the index value of the element in $k^{i}$. $\varphi^{s}$ is a positive number and receives partial support for each iteration. The “first significant jump” rule is used to configure $\varphi^{s}$. Specifically, arrange all of the elements $k^{j}$ in ascending order, then calculate the difference between two adjacent elements to produce $d_{0}$, $d_{1}$, …, and set the condition to detect each difference beginning from $d_{0}$ and seek for the first element, e.g.,$d_{j}>{\left\|k^{i}\right\|}_{\infty }/\left(2h\cdot i\right)$, where h is the kernel size and ${\left\|k^{i}\right\|}_{\infty }$ achieved the maximum value in $k^{i}$. Assigning elements with index values j that meet the criterion to $\varphi^{s}$, leads to an adaptive kernel elaboration process, as each element is penalized less in the iterative phase. The objective function of minimization is then expressed as

(14) $$E(k)=\frac{1}{2}{\left\|\nabla I^{s}⊗\nabla B\right\|}^{2}+\gamma \sum_{j\in \bar{S^{i+1}}}\left|k_{j}\right|$$

## 3. Influences on the Accuracy of Kernel Estimation

The features of images have a massive effect on the estimation results of the kernel. Additionally, because it is not well-posed to solve the problem of the blur kernel and the sharp image at the same time, there are variances in the kernels acquired by different algorithms according to the application of blind image deconvolution methods. This is covered in more detail below in terms of both the kernel estimate principle and its practical application.

According to the introduction in Section 2, the computational principle states that salient edges in the images are first screened before their gradients are employed to determine the kernel. There is not enough gradient of the significant edges to calculate an exact kernel if the image is flatter, has less texture detail, and has more low-frequency information. To extract the kernel, image regions with lots of texture detail are selected.

**Figure 2:**
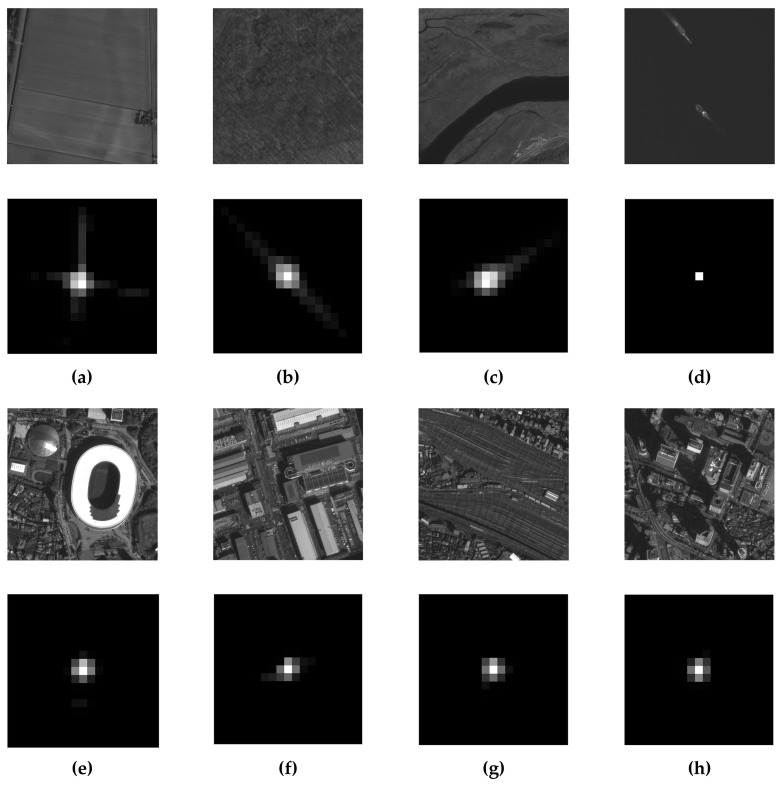
Remote sensing photos from the Jilin-1 and the corresponding kernels: (**a**) Farmland; (**b**) forests; (**c**) river; (**d**) sea; (**e**) gymnasium; (**f**) streets; (**g**) railways; (**h**) buildings.

From the actual extraction effect of the kernel, as shown in Figure 2, where (a) to (d) are farmland, forest, river, and ocean, respectively, the images are flatter and have less high-frequency information, so the kernel is more influenced by the image characteristics. The results of other urban pictures with rich texture features (e) to (h) are more consistent, and the kernels of several images are similar, with minor variations within a given error level.

In summary, the precision of the kernel estimation is influenced by the image characteristics. Typically, the more texture detail an image includes, the better the kernel can be calculated. Although the kernel for different portions of the image is generally stable, it might vary within a specific error range. Moreover, there is no explicit quantitative relationship between the richness of texture detail in an image and the accuracy of the kernel. Therefore, we use the energy concentration (EC) of the kernel to ascertain whether the kernel is valid and refine them to obtain the RIK, which is used to calculate the MTF.

The EC of the kernel indicates the extent to which the energy of the image kernel is aggregated, with a value of

(15) $$EC=\frac{\sum_{pq}D_{pq}^{2}}{\sum_{ij}D_{ij}^{2}},$$

where (p,q) denotes the coordinates of each pixel in the central region of the kernel, (i,j) represents the coordinates of all pixels in the kernel, and D is the corresponding grayscale for each pixel.

## 4. Description of the Process

**Figure 3:**
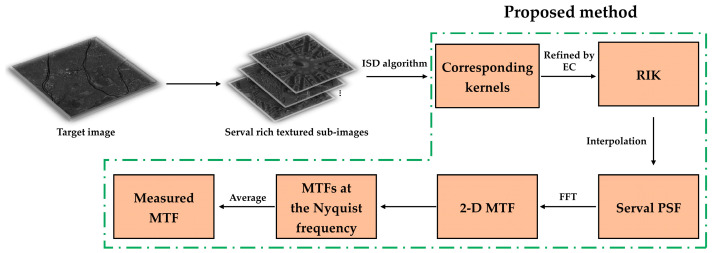
The whole MTF acquisition process.

The flow of the MTF measurement method based on the RIK can be summarized in Figure 3.

The entire method is split into five steps:

1. In the target image, select several sub-images with rich texture details, every 500 × 500 pixels in size;
2. For each sub-image, evaluate the kernel using the principles and computational calculation process given in Section 2.2 of this study;
3. Calculate the central pixel energy concentration of each kernel according to Equation (15). If a discrete value is too high or too low, the related kernel is deemed unreliable and rejected. After refining, the refined image kernels are obtained;
4. The refined image kernel is interpolated to build the PSF, and FFT is performed to obtain the 2-D MTF. The longitudinal and transverse directions are selected to obtain the MTF curves in both directions, and the MTFs at the Nyquist frequency are picked;
5. The final MTF is determined by averaging the MTFs of the two directions from step 4.

## 5. Ground Experimental Results and Analysis

To verify the feasibility and accuracy, the MTF measured by the method presented in this paper was compared with the result of the ISO12233 edge method, which is widely used [13].

### 5.1. Validation Experiment of the MTF Measurement Method

**Figure 4:**
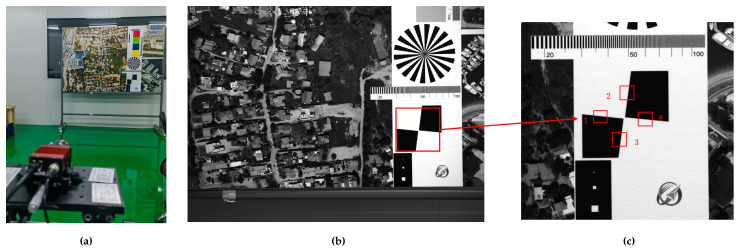
Imaging experiments: (**a**) Diagram of the experimental setup; (**b**) the target scene; (**c**) the knife edge.

Imaging tests were performed in the lab with a high-performance backside-illuminated COMS (Complementary Metal Oxide Semiconductor) camera to confirm the viability of our method. As shown in Figure 4b,c, the target scene and the knife edge are captured simultaneously by the camera, and the image quality is similar across the camera’s fields of view, providing good consistency. Table 1 shows the MTFs at Nyquist frequency in two directions as measured by the ISO12233 edge method, where MTF*_y_* and MTF*_x_* stand for the longitudinal and transverse MTFs, respectively.

**Figure 5:**
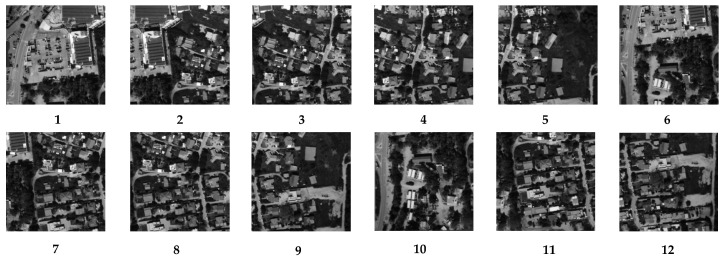
Selected 12 sub-images.

**Figure 6:**
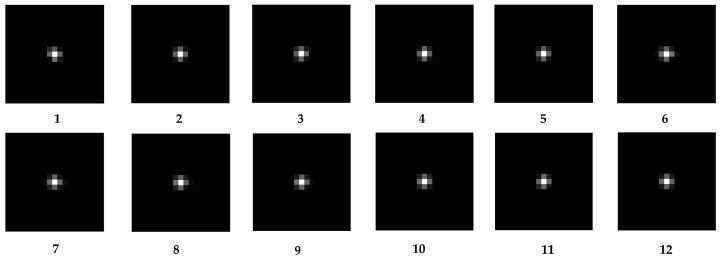
Kernels corresponding to each sub-image.

In following the steps described in Section 4, 12 sub-images with a size of 500 × 500 pixels were chosen, and the kernels were then individually obtained. Figure 5 and Figure 6, respectively, display the sub-images and accompanying kernels. Limited by the experimental conditions, the information contained in the acquired images is not fully available for the precise calculation of the kernel, so the 12 sub-images selected are partially overlapping.

According to Equation (15), the 1 × 1-pixel EC for each kernel is computed (shown in Table 2). The data are all around 0.6 and relatively smooth, with no discrete values that fluctuate significantly. As a result, the kernels of the 12 sub-images can be regarded as suitable for further MTFs calculation. Table 3 illustrates the MTFs at the Nyquist frequency of various orientations obtained by the RIK, and it is obvious that the values are all close to 0.3.

Table 4 displays the longitudinal and transverse average MTFs obtained by two methods, with errors of 6.83% and 1.56%, respectively, when compared to the outcomes obtained by the traditional method, illustrative of the viability of our approach.

### 5.2. The Effect of Image MTF Levels on Measurement Accuracy

Any alteration in the space environment and its internal factors may affect the optical system during the assembly, transportation, launch, and on-orbit operation of satellites, shifting the MTF of the whole system [29]. The measurements may have varied degrees of error with different image MTF levels. To determine how these variations in MTF levels affect measurement accuracy, further research is required.

**Figure 7:**
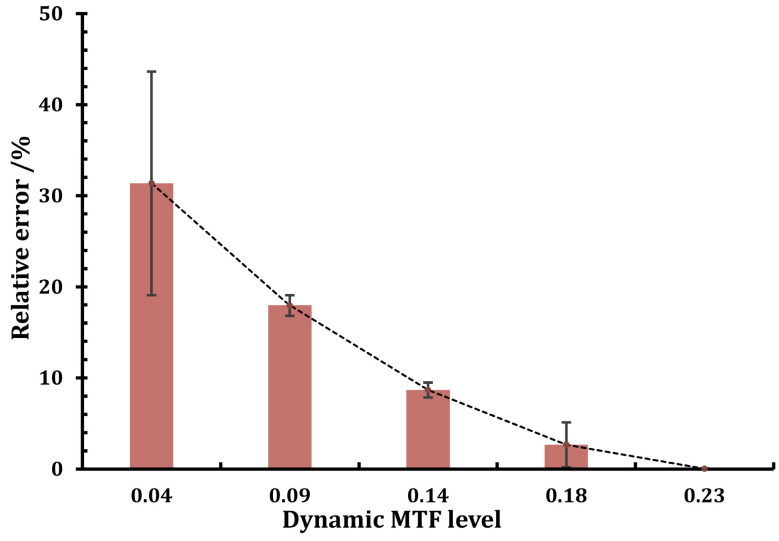
Influence of image MTF level on measurement.

In the lab, various amounts of defocus are applied to the camera to produce groups of images with varying MTF levels. The MTF value is then evaluated using the ISO12233 edge method, and the findings are used as a benchmark. Afterward, we measured the MTF using the method suggested in this paper and obtained the relative error of the measured value for various image MTF levels, as shown in Figure 7.

The figure shows that when the image MTF is at a low level, there is a minor absolute error between the measurement findings and the reference value but a large relative error, making the measurement results at this point untrustworthy. In the latter sets of data, as the image MTF increases, the relative error quickly converges to an acceptable range, so we believe that the results obtained by the method proposed in this paper are generally accurate when the image MTF is greater than 0.1. As far as we know, the dynamic MTF of current on-orbit remote sensing satellites is typically in the range of 0.07 to 0.2 according to the design requirements, and within this range, the method suggested in this paper can meet the precision requirements of on-orbit MTF measurement.

### 5.3. The Influence of Image SNR on MTF Measurement Results

As was discussed in Section 5.1, it can be seen that our method yields satisfactory results in an ideal environment such as the lab; at this time, the SNR of all images captured by the camera is above 40 dB. However, while space imagers are in orbit, the sensor circuitry and exogenous noise brought on by the environment typically perturb them, leading to distorted and degraded remote sensing images [30]. Therefore, it is crucial to investigate how image noise impacts the accuracy of MTF measurement.

**Figure 8:**
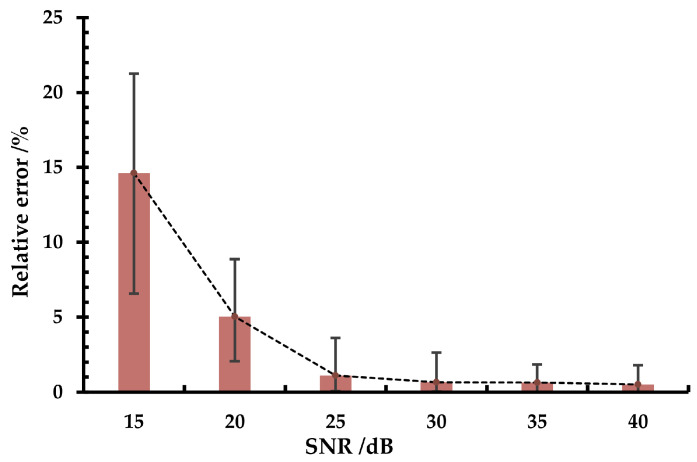
Impact of image noise on MTF measurement results.

The 12 sub-images chosen in the previous part are combined with the noise, then the kernel is obtained, and the MTF is determined. The MTFs obtained from the sub-images with different SNRs are compared with the MTFs in the noise-free case, and the relative error of the MTFs at different SNRs is shown in Figure 8.

It can be seen that at an image SNR of 15 dB, the relative error of the MTF fluctuates around 15% and can reach a maximum of over 20%; in this case, the result is seriously affected by noise. However, as the SNR increases, the relative error converges quickly and is below 5% (mean value). Therefore, these analyses indicate that the method proposed in this paper has good robustness when the image SNR is above 20 dB.

## 6. Application of On-Orbit Satellite MTF Assessment

**Figure 9:**
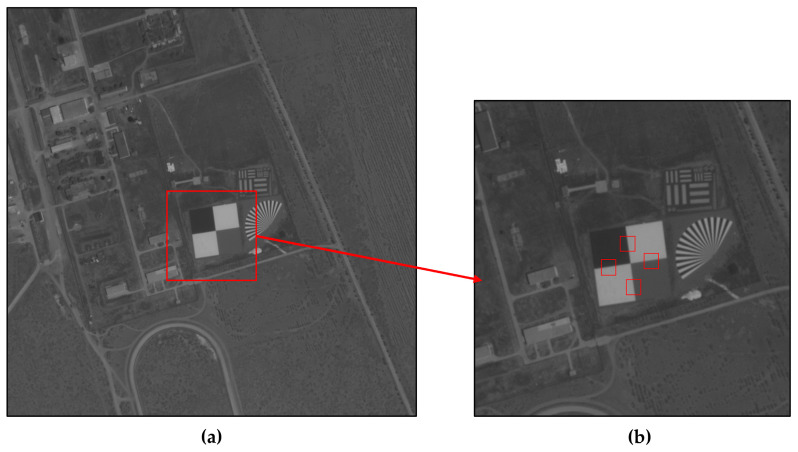
Testing image captured by Jilin-1 satellite: (**a**) Calibration site; (**b**) knife-edge target.

**Figure 10:**
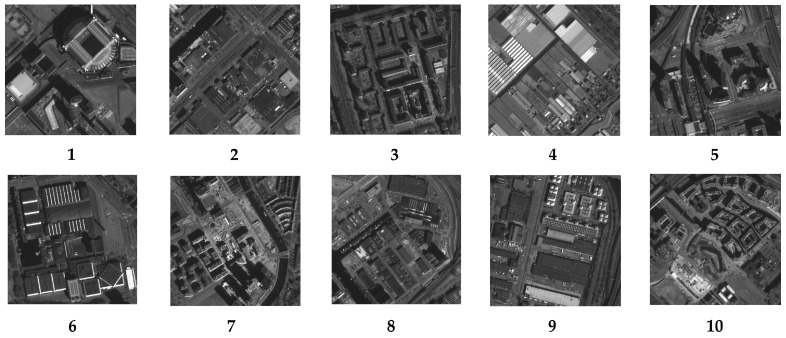
The 10 cloud-free sub-images with rich texture detail picked from the image acquired by Jilin-1 with a size of 500 × 500 pixels.

**Figure 11:**
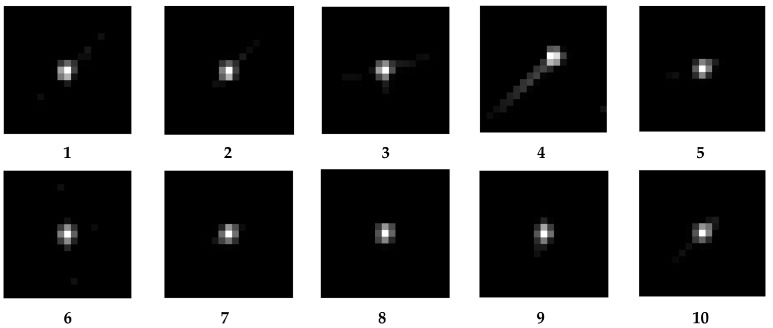
Kernels of the remote sensing sub-images (10 cloud-free sub-images).

As shown in Figure 9, the knife-edge target of the calibration site was employed to test the MTF of the Jilin-1 satellite, and the results are shown in Table 5. This method requires satellites to image a specific location. Due to the restriction of satellite orbit and return visit period, it is difficult to achieve frequent intensive monitoring. Therefore, we attempt to estimate the on-orbit MTF using the method presented in this research. We obtained 10 cloud-free sub-images with rich texture detail picked from the image acquired by Jilin-1 with a size of 500 × 500 pixels (illustrated in Figure 10) and their corresponding kernels (illustrated in Figure 11).

The central pixel EC of each kernel is shown in Table 6. Most of the EC is around 0.45, besides the 4th, which is below 0.3, much lower than the others and should be considered discrete values, so the kernel of the fourth sub-image will be excluded from the subsequent MTF calculation.

After removing sub-image 4, the two directions MTFs at the Nyquist frequency were calculated from the RIK, as shown in Table 7. It is easy to see that the transverse MTF is around 0.15 and 0.25 in the longitudinal, and the average MTFs for each direction are displayed in Table 8. The error of the horizontal and vertical MTF values are 0.14% and 1.38%, respectively, in comparison to the measurement results obtained using the ISO12233 edge method. This excellent agreement further confirms the viability and effectiveness of the optical remote sensing sensors on-orbit MTF measurement method based on the RIK introduced in this paper.

## 7. Conclusions

Based on the drawbacks of traditional MTF measurement methods, this paper proposes an on-orbit MTF measurement method for remote sensing imagers, which is based on RIK instead of natural feature sceneries or artificial knife edges, point sources, and other targets. RIK is given by EC refinement of the image kernel extracted from remote sensing images with rich texture details (such as cities and buildings) directly using the ISD algorithm. Then the PSF is built by interpolating the RIK. Finally, the MTF of the optical system is calculated by the Fourier transform. Compared with the conventional method, the measured MTFs at the Nyquist frequency have an error of no more than 7% in the lab, and the on-orbit MTF of the Jilin-1 satellite measured from remote sensing images with a maximum error of no more than 2%, which proves the feasibility and validity of our method. At image SNR of 20dB~40dB, the average relative error of the measured findings is below 5%, demonstrating the good robustness of this method. This paper contributes an innovative strategy for on-orbit imaging quality assessment of high-resolution remote sensing satellite space optical payloads. On-orbit optical remote sensing satellites can continuously transmit remote sensing images to the ground, and it is easy to find sub-images within these that contain rich details (e.g., buildings, streets, etc.). Therefore, in theory, by using the method proposed in this paper, it is possible to perform high-frequency measurements of the in-orbit MTF of remote sensing satellite optical payloads and to achieve real-time monitoring of their imaging quality.

## Figures and Tables

**Table 1:** MTFs measured by ISO12233 edge method.

	Position	Value	Average
MTF_y_	2	0.275	0.2930
	3	0.311	
MTF_x_	1	0.293	0.2945
	4	0.296	

**Table 2:** The 1 × 1-pixel EC of each sub-image kernel.

**No.**	**1**	**2**	**3**	**4**	**5**	**6**
EC	0.6017	0.6146	0.6040	0.6091	0.6071	0.5663
**No.**	**7**	**8**	**9**	**10**	**11**	**12**
EC	0.5820	0.5923	0.5903	0.5813	0.5903	0.6018

**Table 3:** MTFs obtained from RIK of each sub-image.

No.	MTF_y_	MTF_x_
1	0.30654	0.30689
2	0.29654	0.30514
3	0.28076	0.27833
4	0.29357	0.28511
5	0.28185	0.28691
6	0.23811	0.32868
7	0.24301	0.32773
8	0.25939	0.30106
9	0.24752	0.31009
10	0.23567	0.27820
11	0.28195	0.28091
12	0.27849	0.30007
Standard Deviation	2.45%	1.77%

**Table 4:** Average MTF of the two methods at the Nyquist frequency.

	ISO12233 Edge	Our Method	Error
MTF_y_	0.2930	0.2703	6.83%
MTF_x_	0.2945	0.2991	1.56%

**Table 5:** MTF of the Jilin-1 satellite measured by ISO12233 edge method.

Plan	MTF_y_		MTF_x_	
	MTF_y_-1	MTF_y_-2	MTF_x_-1	MTF_x_-2
57,648	0.240	0.234	0.134	0.168
57,676	0.216	0.243	0.143	0.147
57,784	0.244	0.253	0.132	0.153
Average	0.2383		0.1462	

**Table 6:** The 1 × 1-pixel EC of remote sensing sub-image kernels.

**No.**	**1**	**2**	**3**	**4**	**5**	**No.**
EC	0.3993	0.4480	0.4226	0.2814	0.3993	EC
**No.**	**6**	**7**	**8**	**9**	**10**	**No.**
EC	0.4491	0.4644	0.4721	0.4721	0.4634	EC

**Table 7:** On-orbit MTFs obtained from the RIK of each sub-image.

No.	MTF_y_	MTF_x_
1	0.26577	0.15738
2	0.22907	0.13844
3	0.21845	0.16020
5	0.19790	0.14565
6	0.28322	0.14446
7	0.20729	0.15260
8	0.26953	0.13841
9	0.28901	0.14984
10	0.15477	0.12696
Standard Deviation	4.51%	1.04%

**Table 8:** On-orbit average MTF of the two methods.

	ISO12233 Edge	Our Method	Error
MTF_y_	0.2383	0.2350	1.38%
MTF_x_	0.1462	0.1460	0.14%

## Data Availability

Data are contained within the article.
